# Identifying genetic markers enriched by brain imaging endophenotypes in Alzheimer’s disease

**DOI:** 10.1186/s12920-022-01323-8

**Published:** 2022-08-01

**Authors:** Mansu Kim, Ruiming Wu, Xiaohui Yao, Andrew J. Saykin, Jason H. Moore, Li Shen, for the Alzheimer’s Disease Neuroimaging Initiative

**Affiliations:** 1 Department of Artificial intelligence, Catholic University of Korea, Bucheon, Republic of Korea; 2 School of Engineering and Applied Science, University of Pennsylvania, Philadelphia, USA; 3 Department of Biostatistics, Epidemiology and Informatics, Perelman School of Medicine at the University of Pennsylvania, Philadelphia, USA; 4 Indiana Alzheimer Disease Center and Department of Radiology and Imaging Sciences, Indiana University School of Medicine, Indianapolis, USA; 5 Department of Computational Biomedicine, Cedars Sinai Medical Center, West Hollywood, USA

**Keywords:** Brain imaging genetics, Genome-wide association study, Imaging-diagnosis map, Imaging-genetics map

## Abstract

**Background:**

Alzheimer’s disease (AD) is a complex neurodegenerative disorder and the most common type of dementia. AD is characterized by a decline of cognitive function and brain atrophy, and is highly heritable with estimated heritability ranging from 60 to 80$\%$. The most straightforward and widely used strategy to identify AD genetic basis is to perform genome-wide association study (GWAS) of the case-control diagnostic status. These GWAS studies have identified over 50 AD related susceptibility loci. Recently, imaging genetics has emerged as a new field where brain imaging measures are studied as quantitative traits to detect genetic factors. Given that many imaging genetics studies did not involve the diagnostic outcome in the analysis, the identified imaging or genetic markers may not be related or specific to the disease outcome.

**Results:**

We propose a novel method to identify disease-related genetic variants enriched by imaging endophenotypes, which are the imaging traits associated with both genetic factors and disease status. Our analysis consists of three steps: (1) map the effects of a genetic variant (e.g., single nucleotide polymorphism or SNP) onto imaging traits across the brain using a linear regression model, (2) map the effects of a diagnosis phenotype onto imaging traits across the brain using a linear regression model, and (3) detect SNP-diagnosis association via correlating the SNP effects with the diagnostic effects on the brain-wide imaging traits. We demonstrate the promise of our approach by applying it to the Alzheimer’s Disease Neuroimaging Initiative database. Among 54 AD related susceptibility loci reported in prior large-scale AD GWAS, our approach identifies 41 of those from a much smaller study cohort while the standard association approaches identify only two of those. Clearly, the proposed imaging endophenotype enriched approach can reveal promising AD genetic variants undetectable using the traditional method.

**Conclusion:**

We have proposed a novel method to identify AD genetic variants enriched by brain-wide imaging endophenotypes. This approach can not only boost detection power, but also reveal interesting biological pathways from genetic determinants to intermediate brain traits and to phenotypic AD outcomes.

**Supplementary Information:**

The online version contains supplementary material available at 10.1186/s12920-022-01323-8.

## Background

Alzheimer’s Disease (AD) is a complex neurodegenerative disorder, and the most common type of dementia [1]. Today, approximately 5.8 million people are suffering from AD-related dementia in the United States, and it is expected to exceed 13.8 million by 2050 [2]. AD is characterized by a decline of cognitive function and brain atrophy, and it is highly heritable with estimated heritability ranging from 60 to 80$\%$ [3]. The most straightforward and widely used strategy to identify AD-related genetic markers such as single-nucleotide polymorphisms (SNPs) is to perform genome-wide association studies (GWAS) or GWAS-based meta-analyses on a case-control diagnostic phenotype. Using this strategy, recent studies (e.g., [1, 4, 5]) have identified over 50 AD related susceptibility loci. This method faces a major burden for multiple comparison correction and thus requires a large sample size to detect SNPs with small effect sizes.

To address this challenge, imaging genetics [6] is emerging as a new promising field, where imaging quantitative traits (QTs) are used as phenotypes to identify relevant genetic markers. Of note, imaging QTs are quantitative measures, statistically more powerful than binary case-control status, and thus have greater potential to identify subtle genetic signals with small effect sizes from study cohorts of moderate sample sizes [7]. For example, Shen et al. [8] used cortical thickness, volume, and gray matter density measures as QTs to examine genetic effects in AD. Given that many imaging genetics studies did not involve the diagnostic outcome directly in the analyses, the identified imaging or genetic markers may not be related or specific to disease outcomes such as AD.

To bridge this gap, we propose an innovative method to identify disease-related genetic variants enriched by imaging endophenotypes, which are the imaging traits associated with both genetic factors and disease status. We demonstrate the promise of our method by applying it to the Alzheimer’s Disease Neuroimaging Initiative (ADNI) database [9]. Our major contributions are twofold: (1) We propose a novel approach to identify AD genetic variants enriched by brain-wide imaging endophenotypes. This approach can not only boost detection power, but also reveal interesting biological pathways from genetic determinants to intermediate brain traits and then to phenotypic AD outcomes. (2) We show the effectiveness of our approach in an empirical study to link genetics with three disease outcomes [i.e., early mild cognitive impairment (EMCI), late MCI (LMCI), and AD] via mapping and correlating their associations with region-based amyloid imaging QTs across the brain.

## Results

We first report the results of our first experiment. In this experiment, to demonstrate the promise of our approach, we performed a comparative study with a few conventional genetic association methods. The benchmark algorithms used in this work include: (1) conventional GWAS analysis controlled for relevant covariates including age, sex and education (implemented in PLINK v1.90), (2) Pearson’s correlation analysis between each SNP and each diagnosis outcome, and (3) the partial correlation analysis between each SNP and each diagnosis outcome while controling for relevant covariates. We performed empirical comparison on the real imaging genetics data from the ADNI cohort. On the genetics side, we analyzed 54 AD SNPs identified in recent landmark AD GWAS studies [1, 4, 5]. On the imaging QT side, we analyzed the AV-45 PET data due to its high sensitivity and specificity for distinguishing AD from MCI and CN [10, 11]. Three case-control comparisons (i.e., CN vs EMCI, CN vs LMCI, CN vs AD) were studied to explore imaging genetic effects on different disease stages.

### Identification of genetic variants associated with diagnosis based on GWAS analysis

Conventional GWAS analysis was applied to identify genetic variants for three diagnostic outcomes (i.e., CN vs. AD, CN vs. LMCI, and CN vs. EMCL), respectively. APOE-rs429358 ($\hbox {corrected-}p=6.23\times 10^{-13}$) and PVRL2-rs41289512 ($\hbox {corrected-}p=2.09\times 10^{-4}$) were significantly associated with AD diagnosis, and 2) APOE-rs429358 ($\hbox {corrected-}p=2.51\times 10^{-3}$) was also significantly associated with LMCI diagnosis. No significant SNPs were identified to be associated with EMCI diagnosis. Table 1 shows the detailed results.

**Table 1:**
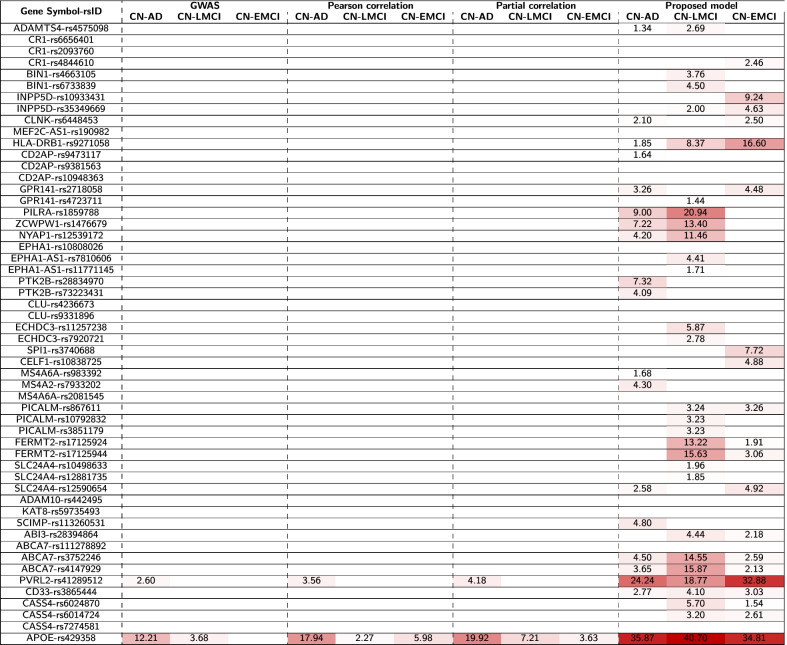
The comparison of identified genetic variants.

We compare the significance of the identified genetic variants using the GWAS, Pearson’s correlation, partial correlation, and our model. Corrected-p values are reported in the format of $-log_{10}(p)$

### Identification of genetic variants associated with diagnosis based on correlation analysis

The Pearson’s correlation and partial correlation analyses were applied to identify genetic variants related to three diagnostic outcomes. These two correlation analyses yielded very similar genetic findings. The detailed results are shown in Tables 1 and 2.

For Pearson’s correlation analysis, we observed that APOE-rs429358 was significantly correlated with all three diagnoses (i.e., $r=0.42$ and $\hbox {corrected-}p=1.15\times 10^{-18}$ for AD diagnosis, $r=0.18$ and $\hbox {corrected-}p=5.40\times 10^{-3}$ for LMCI diagnosis, and $r=0.26$ and $\hbox {corrected-}p=1.04\times 10^{-6}$ for EMCI diagnosis), and PVRL2-rs41289512 was significantly correlated with AD diagnosis ($r=0.22$ and $\hbox {corrected-}p=2.75\times 10^{-4}$).

For partial correlation analysis, we observed that APOE-rs429358 was significantly correlated with all three diagnoses (i.e., $r=0.44$ and $\hbox {corrected-}p=1.20\times 10^{-20}$ for AD diagnosis, $r=0.28$ and $\hbox {corrected-}p=6.15\times 10^{-8}$ for LMCI diagnosis, and $r=0.20$ and $\hbox {corrected-}p=2.32\times 10^{-4}$ for EMCI diagnosis), and PVRL2-rs41289512 was significantly correlated with AD diagnosis ($r=0.23$ and $\hbox {corrected-}p=6.66\times 10^{-5}$).

**Table 2:**
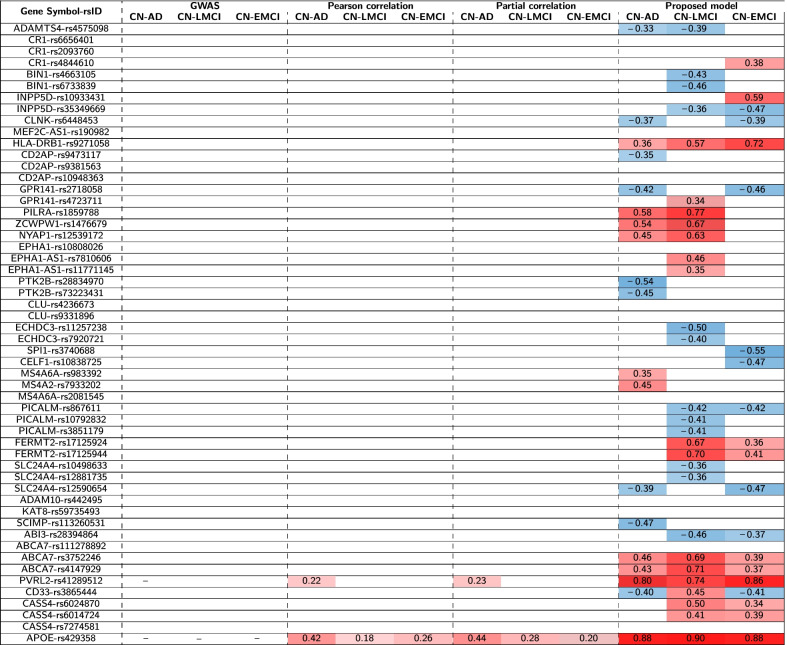
The comparison of the identified genetic variants.

We compare correlation coefficients of identified genetic variants using Pearson’s correlation, partial correlation, and our model. We removed all non-significant genetic variants (corrected-p > 0.05). We also removed GWAS results because it is based on the regression model. Red and blue colors correspond to identified genetic variants with positive and negative correlation coefficients, respectively

### Identification of genetic variants associated with diagnosis via correlating their effect maps on brain

#### Step 1. Imaging-diagnosis association analysis

**Fig. 1:**
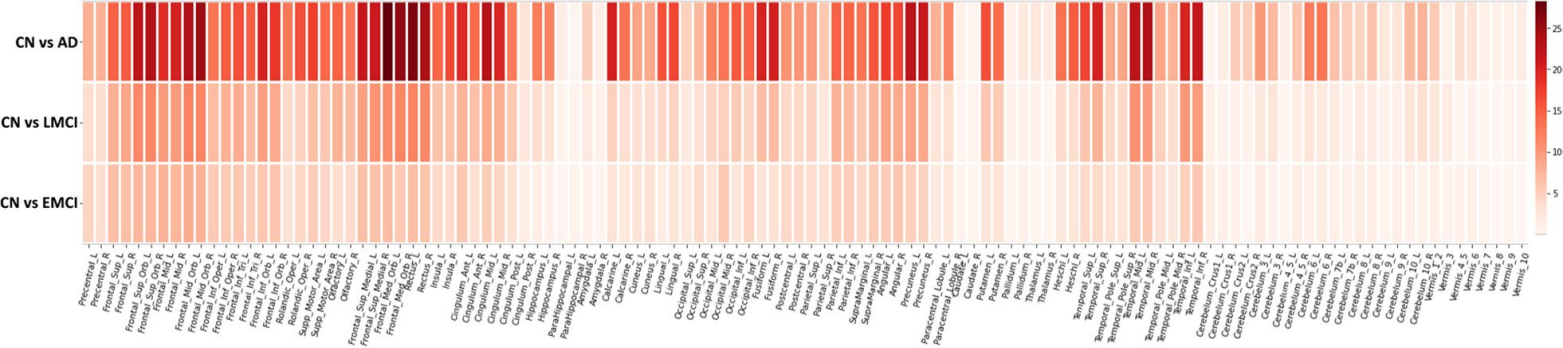
The significance heat map for imaging-diagnosis analysis. The effect of the diagnosis outcome on AV-45 imaging QT data is estimated at each ROI. For each ROI-diagnosis pair, $-log_{10}(p)$ is color-coded and shown in the heat map

The linear regression model was applied to examine the diagnostic effect on AV-45 imaging QTs. Figure 1 shows the resulting p-value maps for three comparisons (i.e., CN vs. EMCI, CN vs. LMCI, CN vs. AD), where $-log_{10}(p)$ values are shown. On average, CN versus AD yielded the most significant diagnostic effects on imaging QTs, and CN versus EMCI yielded the least significant ones. This matches our intuition about the abnormality change of the amyloid imaging QTs over the disease progression. Table 3 shows the top 10 significant regions for the three analyses. Eight regions are common across the three disease stages, including left and right medial orbital superior frontal gyrus, rectus and middle orbital frontal gyrus, right superior orbital frontal gyrus, and left middle temporal gyrus.

**Table 3:** Top 10 significant ROIs from imaging-diagnosis analysis.

Rank	CN versus AD		CN versus LMCI		CN versus EMCI	
	ROI	p-value	ROI	p-value	ROI	p-value
1	Frontal_Med_Orb_L	28.26	Frontal_Mid_Orb_L	11.94	Frontal_Mid_Orb_L	8.31
2	Rectus_L	26.92	Rectus_L	11.83	Frontal_Mid_Orb_R	7.94
3	Frontal_Med_Orb_R	25.86	Frontal_Mid_Orb_R	11.78	Frontal_Mid_L	7.83
4	Frontal_Mid_Orb_R	25.06	Frontal_Med_Orb_R	11.63	Frontal_Mid_R	7.45
5	Rectus_R	23.87	Frontal_Sup_Orb_R	11.57	Frontal_Sup_Orb_R	7.36
6	Temporal_Mid_R	23.48	Frontal_Sup_Orb_L	11.47	Frontal_Sup_Orb_L	7.32
7	Frontal_Mid_Orb_L	23.39	Rectus_R	11.38	Frontal_Med_Orb_L	6.77
8	Frontal_Sup_Orb_R	23.37	Frontal_Med_Orb_L	11.12	Rectus_L	6.75
9	Cingulum_Mid_L	23.16	Temporal_Mid_L	10.55	Frontal_Sup_R	6.70
10	Temporal_Mid_L	22.73	Frontal_Sup_Medial_R	10.39	Frontal_Sup_L	6.69

We examine the spatial effect of diagnosis outcomes (i.e., CN vs. AD, CN vs. LMCI, CN vs. EMCI) on the Av-45 imaging data. The significant level of the diagnostic effect on the ROI is reported in the format of $-log_{10}(p)$

#### Step 2. Imaging-genetics association analysis

We performed an univariate imaging genetics analysis to examine genetic effects of each studied SNP on each AV-45 imaging QT. Figure 1 shows the resulting p-value maps for three groups (i.e., CN vs. EMCI, CN vs. LMCI, CN vs. AD), where $-log_{10}(p)$ values are shown. The imaging genetic patterns appear to be similar among these three groups, while CN versus AD and CN versus LMCI yielded slightly stronger imaging genetic associations than CN versus EMCI. In all three cases, APOE-rs429358 and PVRL2-rs41289512 have significant effects on most of the ROIs. Specifically, most of the 116 brain ROIs (i.e., 97 ROIs for CN vs. AD, 98 ROIs for CN vs. LMCI, and 89 ROIs for CN vs. EMCI) were significantly associated with APOE-rs429358. The PVRL2-rs41289512 had significant genetic effects on 79 ROIs for CN versus AD, 68 ROIs for LMCI versus AD, and 65 ROIs for CN versus EMCI. The full list of SNP-ROI findings for the three comparisons are available in Additional file 1: Table S1.

#### Step 3. Correlation analysis between brain maps of diagnostic effect vs genetic effect

After estimating genetic and diagnostic effects on the AV-45 imaging QTs, we performed the Pearson’s correlation analysis to identify the genetic variants associated with the diagnosis outcomes via correlating the genetic and diagnostic maps in the brain. In Tables 1 and 2, we observed that APOE-rs429358 obtained the highest correlations with all three diagnoses (i.e., $r=0.88$ and $\hbox {corrected-}p=1.35\times 10^{-36}$ for AD diagnosis, $r=0.90$ and $\hbox {corrected-}p=1.99\times 10^{-41}$ for LMCI diagnosis, and $r=0.88$ and $\hbox {corrected-}p=1.53\times 10^{-35}$ for EMCI diagnosis), and PVRL2-rs41289512 obtained the second highest correlations (i.e., $r=0.80$ and $\hbox {corrected-}p=5.82\times 10^{-25}$ for AD diagnosis, $r=0.74$ and $\hbox {corrected-}p=1.72\times 10^{-19}$ for LMCI diagnosis, and $r=0.86$ and $\hbox {corrected-}p=1.33\times 10^{-33}$ for EMCI diagnosis). According to Tables 1 and 2, we observed that our newly proposed method identified a lot more significant SNPs in all three comparisons than the convectional GWAS and Pearson’s correlation and partial correlation analyses. This shows the promise of our method, which identifies SNP-diagnosis associations through mapping their effects on the imaging QTs across the brain. Many of these QTs can serve as endophenotypes linking genetic factors to disease outcomes. This approach can not only boost detection power, but also reveal interesting biological pathways from genetic determinants to intermediate brain traits and to phenotypic AD outcomes.

### Comparison with analyzing “non-AD” SNPs

**Fig. 2:**
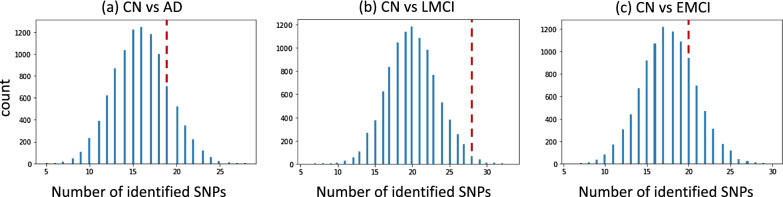
The distribution of the number of SNPs identified from applying the proposed pipeline to randomly selected 54 SNPs (across 10,000 runs).** a**–**c** show the histograms of the number of SNPs identified for three different diagnostic comparisons (i.e., CN vs. AD, CN vs. LMCI, and CN vs. EMCI) respectively. The red dashed line indicates the number of SNPs identified from the pipeline using 54 AD susceptibility loci

Now we report the results of our second experiment, which is designed to compare the findings between the analysis of the above 54 AD susceptibility loci versus the analysis of a same number of random “non-AD” SNPs that have not been linked to AD before. As mentioned in the “Methods” section, we ran our pipeline 10,000 times, and each time it was applied to 54 randomly selected “non-AD” SNPs. Figure 2 shows the distribution of the number of significant findings across these 10,000 analyses on the random “non-AD” SNPs. For comparison purposes, the number of significant findings from analyzing the 54 AD susceptibility loci is plotted as a red dash line in Fig. 2; and the details of these findings are shown in Table 1. Specifically, using this pipeline, our analysis on 54 AD susceptibility loci yielded 19 findings for the CN versus AD comparison, which outperformed 86.88% analyses on the random “non-AD” SNPs (Fig. 2a); yielded 28 findings for the CN vs LMCI comparison, which outperformed 99.35% analyses on the random “non-AD” SNPs (Fig. 2b); and yielded 20 findings for the CN versus EMCI comparison, which outperformed 81.45% analyses on the random “non-AD” SNPs (Fig. 2c). These findings indicate that our pipeline has a higher probability to identify AD susceptibility loci than random SNPs that have not yet been linked to AD before. Of note, our pipeline is not designed to directly examine SNP-diangosis associations. However, the above observation demonstrates the promise of our proposed strategy to identify AD-related SNPs through mapping brain imaging endophenotypes.

## Discussion

A major challenge in AD genetics is how to effectively detect weak signals such as SNPs with small effect sizes. Various strategies have been proposed to increase detection power in genetic association studies [6]. For example, one approach could be to focus on analyzing a small number of prioritized SNPs with relevant functional annotation (e.g., those from the amyloid pathway, or expression QT loci related to brain tissues) to reduce burden for multiple comparison correction. Another approach could be to use enrichment analysis to look for stronger collective effects at the pathway or network level instead of the individual effect from each single SNP.

**Fig. 3:**
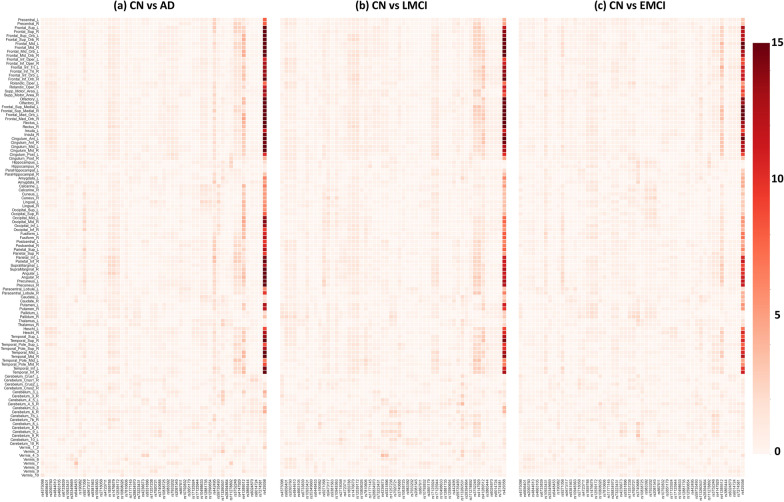
The significance heat map for imaging-genetics analysis. Sub-figures (**a**), (**b**), (**c**) show the p-value significance of imaging-genetics analysis for all SNP-ROI pairs on three data sets (i.e., CN vs. AD, CN vs. LMCI, and CN vs. EMCI), respectively. For each ROI-SNP pair, $-log_{10}(p)$ is color-coded and shown in the heat map

In this work, we have proposed an innovative pipeline to identify interesting SNP-disease associations supported or enriched by the intermediate imaging QTs (endophenotypes) across the entire brain. To the best of our knowledge, this is the first approach to enrich genetic variants associated with AD via mapping their association patterns with imaging QTs in the brain. In our empirical study on the ADNI data, we confirmed multiple genetic variants estimated by conventional models, such as APOE ($\hbox {corrected-}p=1.35\times 10^{-36}$, rs429358) and PVRL2 ($\hbox {corrected-}p=5.82\times 10^{-25}$, rs41289512), as well as other AD-related genetic variants shown in Tables 1 and 2. Furthermore, we demonstrated our analysis could identify a lot more SNPs than conventional approaches. In addition, the significant imaging QTs identified in Figs. 1, 2, 3 have the potential to serve as imaging endophenotypes linking the genetic variant with the disease outcome.

The traditional strategy for identifying genetic variants related to diagnosis is GWAS analysis, which tests the effect of SNP on the diagnostic outcome. Although it has been widely used, a conventional GWAS analysis does not have enough power to identify weak signals from moderately sized study samples. Joint mapping of SNP and diagnostic effects via neuroimaging data, which provide quantifiable traits of disease, can potentially reveal new insights to identify weak but meaningful endophenotype-backed genetic signals for the disease outcome. Compared to traditional strategy, our approach obtained much stronger signals of significance as well as correlation coefficients and identified a lot more interesting genetic variants missed by traditional methods, as shown in Table 1. For example, our approach yielded a much more significant APOE-rs429358 signal than traditional methods. Even though all 54 SNPs have been associated with AD in prior large-scale GWAS studies, applying traditional methods to our moderately sized ADNI sample only yielded two significant SNPs. However, using the proposed method, we obtained 41 significant SNPs. These findings suggest that the proposed method has the potential to boost the detection power.

In our analysis, the following 16 SNPs were found significantly correlated with two or three diagnostic outcomes: rs4574098, rs35349669, rs6448453, rs2718058, rs1859788, rs1476679, rs12539172, rs867611, rs17125924, rs17125944, rs12590654, rs28394864, rs28394864, rs6014724 and rs6024870. Furthermore, the following five SNPs were significant and positively correlated with all diagnosis outcomes: rs9271058, rs3752246, rs4147929, rs41289512, and rs429358. These identified SNPs have the same sign of correlation coefficients. Our findings are in accordance with previous studies [12–20]. For example, rs9271058 was associated with higher expression levels of HLA-DRB1 in various brain regions [15]. The rs3752246 G allele carriers were observed to have an increased risk for developing AD considering C/C genotype as reference category [21]. A recent study showed that the rs4147929 variant minor A allele could significantly increase ABCA7 expression, and ABCA7 showed significantly increased gene expression in AD patients compared with controls [22]. These findings demonstrate the effectiveness of our model on identifying biologically meaningful genetic findings.

Interestingly, we found that only one SNP (i.e., CD33, rs3865444) reported mixed signs of correlation coefficients (i.e., -0.40 for AD diagnosis, 0.45 for LMCI diagnosis, -0.41 for EMCI diagnosis). A previous study showed a bidirectional modulation of CD33 genotype associated cognitive performance [23]. The CD33 genotype had a positive correlation with cognitive function than the CD33 when the precuneus gFCD is lower than 0.04 a. However, the relationship became negative when gFCD in precuneus increased to 0.58. With these observations, it warrants further investigation on independent cohorts to have a better understanding of the mixed directionality on the association between CD33 rs3865444 and different diagnostic stages.

In our second experiment, we performed comparative studies through applying the proposed pipeline to random “non-AD” SNPs. It is promising that our pipeline has been able to to identify a lot more AD susceptibility loci than random SNPs that have not yet been linked to AD before. However, these random SNP analyses have also yielded some significant findings, which warrant further investigation towards a few interesting directions. First, some of these findings could be true signals missed by the existing studies (e.g., due to small effect sizes). Thus, our findings could provide valuable guidance for subsequent replication studies in independent cohorts. Second, some of the findings may not have a direct effect on the diagnosis. However, by the design of our pipeline, these findings are indirectly connected to the diagnostic phenotype via being related to a same set of imaging endophenotypes. Such a mechanism warrants a more detailed further investigation. Third, some of these findings could be false discoveries. One potential limitation of our method is that we include all the ROIs in the brain while correlating the genetic map with the diagnostic map. It is likely that some ROIs are irrelevant to the pathway from genetics to phenotypical outcomes. They may introduce noises and biases, leading to possible false discoveries. Thus, an interesting future direction could be to identify only relevant ROIs for mapping genetic and diagnostic effects. Another interesting direction could be to explore different and improved mapping strategies. For example, the current pipeline employs a linear regression model in both Step 1 and Step 2. This simple modeling strategy coupled with the existence of irrelevant ROIs could lead to false positive or negative discoveries. Expanding to a nonlinear model, such as polynomial regression or a fully connected neural network model, has the potential to capture complex associations and improve biomarker identification. This remains as an interesting future topic to explore.

## Conclusion

We have proposed an innovative method to identify disease-related genetic variants enriched by imaging endophenotypes, which are the imaging quantitative traits (QTs) associated with both genetic factors and disease status. Our approach consists of three steps: (1) association analysis between imaging QTs and diagnosis, (2) association analysis between imaging QTs and each genetic variant, and (3) correlation analysis between two brain maps produced in Step 1 (i.e., map of diagnostic effect) and Step 2 (i.e., map of genetic effect). We applied our method to the ADNI cohort to identify genetic markers enriched by amyloid imaging endophenotypes in AD. Among 54 AD related susceptibility loci reported in prior large-scale AD GWAS, our approach identified 41 of those from a much smaller study cohort (i.e., ADNI) while the standard genetic assocation approaches identified only two of those. Our method yielded not only a lot more AD genetic variants undetectable using the traditional method but also a set of imaging QTs significantly associated with both the genetic variant and the diagnostic outcome. Such QTs have the potential to serve as imaging endophenotypes linking genetics with AD outcomes. These promising findings demonstrate that our approach can not only boost detection power, but also provide valuable information for revealing interesting biological pathways from genetics to brain traits and to AD outcomes. An interesting future topic is to perform an in-depth investigation to explore the detailed relationships between the identified genetic markers and brain imaging traits for different disease stages.

## Methods

### Data description

Data used in the preparation of this article were obtained from the ADNI database (adni.loni.usc.edu) [9]. The ADNI was launched in 2003 as a public-private partnership, led by Principal Investigator Michael W. Weiner, MD. The primary goal of ADNI has been to test whether serial magnetic resonance imaging (MRI), positron emission tomography (PET), other biological markers, and clinical and neuropsychological assessment can be combined to measure the progression of MCI and early AD. For up-to-date information, see www.adni-info.org. In this work, participants (N=971) include 202 AD, 218 LMCI, 296 EMCI, and 255 cognitively normal (CN) subjects with complete baseline data including [$^{18}$F]florbetapir (AV-45) PET scans (measuring amyloid burden), genotyping data, demographic information, and clinical assessments downloaded from the ADNI database (adni.loni.usc.edu). Demographic and clinical assessments of the participants are shown in Table 4.

**Table 4:** Demographic information

	CN	EMCI	LMCI	AD	Total
Number of subject	255	296	218	202	971
Age	76.35 ± 6.54	71.78 ± 7.28	74.71 ± 8.39	75.85 ± 7.67	74.48 ± 7.67
Sex (Male/Female)	132/123	167/129	129/89	123/79	551/420
Education (Year)	16.37 ± 2.64	12.12 ± 2.64	16.12 ± 2.94	15.83 ± 2.81	16.13 ± 2.75

### Data preprocessing

Preprocessed AV-45 PET scans are collected and aligned to the Montreal Neurological Institute space as $2\times 2\times 2$ mm voxels. Standard uptake value ratio is computed by intensity normalization based on a cerebellar crus reference region. We then extract regional neuroimaging measurements from 116 regions-of-interests (ROIs) based on the automated anatomical labeling (AAL) atlas. The genotyping data are downloaded and analyzed using PLINK v1.90 [24]. We perform quality control using the following criteria: genotyping call rate $> 95\%$, minor allele frequency $> 5\%$, and Hardy Weinberg Equilibrium $> 1.00 \times 10^{-6}$. Then, we select 54 susceptibility loci identified by recent AD GWAS or GWAS meta-analysis [1, 4, 5]. The full list of susceptibility loci are shown in Table 5. In addition, we also perform some comparison analyses on “non-AD” related SNPs, which are randomly selected from loci with p-values larger than 0.05 using the summary statistics in a recent landmark AD GWAS study [1].

**Table 5:** Selected AD-related SNPs.

rs-ID	Chromosome	Position	Gene Symbol	rs-ID	Chromosome	Position	Gene Symbol
rs4575098	chr1	161155392	ADAMTS4	rs7920721	chr10	11720308	ECHDC3
rs6656401	chr1	207692049	CR1	rs3740688	chr11	47380340	SPI1
rs2093760	chr1	207786828	CR1	rs10838725	chr11	47557871	CELF1
rs4844610	chr1	207802552	CR1	rs983392	chr11	59923508	MS4A6A
rs4663105	chr2	127891427	BIN1	rs7933202	chr11	59936926	MS4A2
rs6733839	chr2	127892810	BIN1	rs2081545	chr11	59958380	MS4A6A
rs10933431	chr2	233981912	INPP5D	rs867611	chr11	85776544	PICALM
rs35349669	chr2	234068476	INPP5D	rs10792832	chr11	85867875	PICALM
rs6448453	chr4	11026028	CLNK	rs3851179	chr11	85868640	PICALM
rs190982	chr5	88223420	MEF2C-AS1	rs17125924	chr14	53391680	FERMT2
rs9271058	chr6	32575406	HLA-DRB1	rs17125944	chr14	53400629	FERMT2
rs9473117	chr6	47431284	CD2AP	rs10498633	chr14	92926952	SLC24A4
rs9381563	chr6	47432637	CD2AP	rs12881735	chr14	92932828	SLC24A4
rs10948363	chr6	47487762	CD2AP	rs12590654	chr14	92938855	SLC24A4
rs2718058	chr7	37841534	GPR141	rs442495	chr15	59022615	ADAM10
rs4723711	chr7	37844263	GPR141	rs59735493	chr16	31133100	KAT8
rs1859788	chr7	99971834	PILRA	rs113260531	chr17	5138980	SCIMP
rs1476679	chr7	100004446	ZCWPW1	rs28394864	chr17	47450775	ABI3
rs12539172	chr7	100091795	NYAP1	rs111278892	chr19	1039323	ABCA7
rs10808026	chr7	143099133	EPHA1	rs3752246	chr19	1056492	ABCA7
rs7810606	chr7	143108158	EPHA1-AS1	rs4147929	chr19	1063443	ABCA7
rs11771145	chr7	143110762	EPHA1-AS1	rs41289512	chr19	45351516	PVRL2
rs28834970	chr8	27195121	PTK2B	rs3865444	chr19	51727962	CD33
rs73223431	chr8	27219987	PTK2B	rs6024870	chr20	54997568	CASS4
rs4236673	chr8	27464929	CLU	rs6014724	chr20	54998544	CASS4
rs9331896	chr8	27467686	CLU	rs7274581	chr20	55018260	CASS4
rs11257238	chr10	11717397	ECHDC3	rs429358	chr19	45411941	APOE

These include 54 susceptibility loci identified by recent landmark AD genetic studies [1, 4, 5]

### Proposed pipeline for linking SNPs with diagnosis via mapping their regional associations with amyloid imaging QTs across the brain.

The proposed pipeline aims to identify genetic markers enriched by amyloid imaging endophenotypes in AD. The pipeline consists of three steps: (1) association analysis between imaging QTs and diagnosis, (2) association analysis between imaging QTs and each genetic variant, and (3) correlation analysis between two brain maps produced in Step 1 (i.e., map of diagnostic effect) and Step 2 (i.e., map of genetic effect).

#### Step 1. Imaging-diagnosis association analysis

Let *x* be a diagnostic outcome (i.e., case vs. control) and *Y* be a set of AV-45 imaging QTs. We perform the following simple linear regression model to examine the diagnostic effect on each imaging QT $y \in Y$.

1 $$\begin{aligned} y = \beta x + \Gamma Z + \epsilon , \end{aligned}$$

where $Z=(z_1,\ldots ,z_k)^T$ includes the variables whose effects we want to exclude, such as age, sex, and education; $\beta$ and $\Gamma = (\gamma _1,\ldots ,\gamma _k)$ are the coefficients; and $\epsilon$ is the error term. Our goal is to estimate $\beta$ and also test if the diagnosis *x* has a significant effect (i.e. $\beta \ne 0$) on each QT $y \in Y$. As a result, we generate an ROI-based p-value map to quantify the significance of diagnostic effect on imaging data. In this work, each element of the significance map records the “negative log p-value” $-log_{10}(p)$ at the corresponding ROI.

#### Step 2. Imaging-genetics association analysis

Let *G* be a set of SNPs and *Y* be a set of AV-45 imaging QTs. We perform a linear regression model to estimate the additive effect of each SNP $g \in G$ on each QT $y \in Y$. The analysis is performed for all possible SNP-QT pairs, and thus is repeated $54 \times 116 = 6,264$ times. The linear regression model is defined as follows.

2 $$\begin{aligned} y = \alpha g + \Gamma Z + \epsilon , \end{aligned}$$

where $Z=(z_1,\cdots ,z_k)^T$ includes the variables whose effects we want to exclude, such as age, sex, and education; $\alpha$ and $\Gamma = (\gamma _1,\cdots ,\gamma _k)$ are the coefficients; and $\epsilon$ is the error term. Our goal is to estimate $\alpha$ and also test if the SNP *g* has a significant effect (i.e. $\alpha \ne 0$) on each QT $y \in Y$.

Thus, we generate an ROI-based p-value map to quantify the significance of genetic effects on imaging data. Specifically, in this work, each element of the significance map records the “negative log p-value” $-log_{10}(p)$ at the corresponding ROI.

#### Step 3. Correlation analysis between two brain maps (i.e., diagnostic effect vs genetic effect)

In this step, the correlation analysis is applied to score the similarity between two significance maps generated in Steps 1-2. Specifically, Step 1 results in a brain map of the significance level for diagnostic effects in the format of $-log_{10}(p)$, and Step 2 results in multiple brain maps (one for each SNP) containing the significance level for genetic effects in the format of $-log_{10}(p)$. We perform Pearson’s correlation analysis between these two maps to score their similarity. To identify significant correlations, we employ the Bonferroni method to correct for multiple comparison.

### Empirical study on the ADNI data

We conduct an empirical study on the ADNI data to evaluate the promise of the proposed pipeline for identifying novel SNPs related to AD. Our study includes two experiments. In the first experiment, we perform a targeted analysis on 54 AD susceptibility loci (see Table 5) using the proposed pipeline. We compare our findings with those derived from conventional genetic association methods. In the second experiment, we perform a comparative study exploring a same number of randomly selected “non-AD” SNPs that have not yet been linked to AD previously. Specifically, we randomly select 54 “non-AD” SNPs (i.e., $p>0.05$) based on the summary statistics of a landmark AD genetics study [1], apply our pipeline to this SNP set, and report the number of significant findings. We repeat the above analysis 10,000 times with a different set of 54 randomly selected “non-AD” SNPs in each analysis, and report the distribution of the number of significant findings across all these analyses. We compare the number of significant findings from analyzing 54 AD susceptibility loci (see Table 5) in the first experiment with those from analyzing random “non-AD” SNPs in the second experiment.

## Supplementary Information

**Additional file 1.** Supplementary Table S1: The p-value significance for imaging-genetics analysis. Sub-tables (**a**), (**b**), (**c**) show the p-value significance of imaging-geneticsanalysis for all SNP-ROI pairs on three data sets (i.e., CN vs AD, CN vsLMCI, and CN vs EMCI), respectively. For each ROI-SNP pair, the *p*-value is shown.

## Data Availability

The datasets used and analyzed during the study are available in the ADNI LONI repository, https://adni.loni.usc.edu/

## Abbreviations

AD: Alzheimer’s disease
GWAS: Genome-wide association study
SNP: Single-nucleotide polymorphism
ADNI: Alzheimer’s Disease Neuroimaging Initiative
QT: Quantitative trait
EMCI: Early mild cognitive impairment
LMCI: Late mild cognitive impairment
CN: Cognitively normal
